# Evidence-based endoscopic management of Barrett’s esophagus

**DOI:** 10.1093/gastro/gou059

**Published:** 2014-09-17

**Authors:** Patrick Yachimski, Chin Hur

**Affiliations:** ^1^Division of Gastroenterology Hepatology & Nutrition, Vanderbilt University Medical Center, Nashville, TN, USA ^2^Division of Gastroenterology & Institute for Technology Assessment, Massachusetts General Hospital, Harvard Medical School, Boston, MA, USA

**Keywords:** Barrett’s esophagus, esophageal adenocarcinoma, endoscopic surveillance, endoscopic eradication therapy

## Abstract

Barrett’s esophagus (BE) develops as a consequence of chronic esophageal acid exposure, and is the major risk factor for esophageal adenocarcinoma (EAC). The practices of endoscopic screening for—and surveillance of—BE, while widespread, have failed to reduce the incidence of EAC. The majority of EACs are diagnosed in patients without a known history of BE, and current diagnostic tools are lacking in their ability to stratify patients with BE into those at low risk and those at high risk for progression to malignancy. Nonetheless, advances in endoscopic imaging and mucosal therapeutics have provided unprecedented opportunities for intervention for BE, and have vastly altered the approach to management of BE-associated mucosal neoplasia.

## INTRODUCTION

Fundamental paradigm shifts in the endoscopic management of Barrett’s esophagus (BE) have emerged over the past decade. New epidemiologic data have provided revised estimates of the incidence of esophageal adenocarcinoma (EAC) in individuals with BE, prompting re-evaluation of the effectiveness of endoscopic screening and surveillance strategies. At the same time, endoscopic imaging technologies capable of enhanced detection of dysplasia, coupled with safe and effective endoscopic eradication therapies, have expanded therapeutic options for BE and mucosal neoplasia. BE containing high-grade dysplasia (HGD) and/or T1a cancer, formerly treated by surgical esophagectomy, may now in many instances be treated endoscopically, with high expectation of durable remission and cancer-free survival.

## DIAGNOSIS AND SCREENING

EAC is the fourth most common gastrointestinal malignancy. Males are at higher risk than females, and Caucasians are at higher risk than African-Americans. BE is the principal known risk factor for EAC. BE develops as a consequence of chronic exposure to gastric and/or duodenal reflux. While pharmacological therapy for gastroesophageal reflux disease (GERD) exists in the form of histamine receptor antagonists or proton pump inhibitors targeted at gastric acid production, exposure to esophageal bile acid arising from duodenal refluxate may induce a unique esophageal response to injury and also contribute to esophageal carcinogenesis [1].

The classic definition of BE comprises the presence of columnar epithelium with prominent goblet cells indicative of intestinal metaplasia (IM) populating the tubular esophagus proximal to the anatomic squamocolumnar junction. In the West, the distal esophagus is recognised as the top of the gastric folds; in Japan, however, the palisade vessels serve as this landmark. Moreover, whether or not columnar esophageal epithelium without IM qualifies as BE is a point of some debate. On the one hand, the presence of IM is requisite for the diagnosis of BE based on current American Gastroenterological Association (AGA) guidelines [2]. On the other, given the observation that columnar epithelium without IM appears to embody increased cancer risk, current British Society of Gastroenterology guidelines do not require the presence of IM for diagnosis of BE in endoscopically obtained biopsies from the tubular esophagus [3].

Endoscopic screening for BE among individuals with symptomatic GERD has been justified on the basis of cost–effectiveness analyses. In simulated disease models under certain conditions, a one-time endoscopic screening examination among individuals with symptomatic GERD, at age either 50 or 60, may be cost-effective at an acceptable threshold relative to a no-screening strategy [4, 5]; however, restricting screening to individuals with symptomatic GERD fails to account for a considerable pool of asymptomatic individuals who are at risk. In one study of healthy individuals invited to undergo esophago-gastroduodenoscopy at the same time as elective colonoscopy, BE was detected in 8.3% of individuals reporting any heartburn, y*et al.*o in 5.6% of individuals reporting no history of symptomatic heartburn [6]. Emphasizing the potential limitations of a symptom-targeted screening strategy, data from the Northern Ireland Barrett’s Esophagus Register indicate that, among incident cases of EAC, only a minority (7%) arose in individuals with a known pre-existing diagnosis of BE [7]. In this cohort, the ‘absence' of reflux symptoms at time of BE diagnosis appeared to be associated with an increased risk of malignant progression during the surveillance period [8].

In a study by Rubenstein and colleagues, the rationale for endoscopic screening for BE among patients with GERD was examined in the context of other commonly accepted cancer screening strategies, such as colonoscopy for colorectal cancer screening and mammography for breast cancer. When considering age-adjusted incidence of EAC, a screening endoscopy may be warranted in white males over the age of 60 with weekly GERD symptoms. Yet in this analysis, such individuals were as likely to develop breast cancer as EAC—implying that, if an endoscopy for EAC screening is felt to be indicated, one could just as easily justify a mammogram to screen for male breast cancer. In this analysis, the age-adjusted incidence of EAC among women up to the age of 80 never reached a threshold sufficient to warrant screening endoscopy [9].

At present, there are neither retrospective nor prospective clinical data to suggest that endoscopic screening for BE improves early cancer diagnosis or reduces EAC-related mortality. Current AGA guidelines acknowledge the limitations of endoscopic screening, albeit in somewhat measured fashion, recommending against screening for BE among the general population with GERD, while suggesting the screening of individuals with risk factors including age >50 years, male sex, white race, chronic GERD symptoms, and elevated body mass index or abdominal fat distribution [2].

## DISEASE PROGRESSION AND ENDOSCOPIC SURVEILLANCE

A simulated model analysis, published in 1994 by Provenzale and colleagues and assessing various surveillance strategies followed by esophagectomy for HGD or cancer, demonstrated that, from a cost-effectiveness standpoint, a strategy of endoscopic surveillance every 5 years was acceptable (compared with no surveillance) [10]. A surveillance interval of every 5 years was supported as cost-effective in an updated 1999 version of this model, comparing favorably with accepted colon, breast, and cervical cancer screening practices [11]. In both cases, the models were sensitive to estimates of cancer risk. Supported by data from such models, endoscopic surveillance of non-dysplastic BE is still recommended by professional societies including the AGA (3–5 year intervals) [2] and American College of Gastroenterology (3 year intervals) [12]; however, a subsequent model suggested that surveillance was only cost-effective in individuals found to have dysplasia at the index endoscopy [4].

Two critical observations account for the obstacles faced by endoscopic surveillance programs: (i) progression rates to EAC among patients with BE appear to be lower than previously estimated and (ii) mortality among individuals with BE is dominated by etiologies other than EAC.

A frequently cited rate of progression from BE to EAC, of 0.5% per year, was based on an analysis designed to assess the presence of publication bias in the reporting of risk of EAC [13]. More recent epidemiological investigation from national registries has reported considerably lower incidence rates. A Netherlands registry reported a progression rate of 3.0 per 1000 person-years (0.30%) among individuals without dysplasia [14]. From a combined cancer and pathology registry data in Denmark, an even lower incidence rate of 1.2 per 1000 person-years (0.12%) was reported after excluding cases of prevalent cancer [15].

EAC-related mortality is increased in individuals with BE. One recent meta-analysis of nineteen studies reported that EAC was the cause of death in 7% of individuals with BE, yet the majority of deaths in the cohort (>50%) were due to cardiovascular or pulmonary disease. And individuals with BE were more than twice as likely to die due to a non-esophageal malignancy than due to EAC [16]. Also, in the Northern Ireland Barrett’s Esophagus Register, EAC-related mortality was increased among individuals with BE, compared with the general population, but accounted for only 4.7% of deaths in the cohort—and overall mortality among individuals with BE did not differ from that of the general population [17].

With these factors in mind, there are neither retrospective nor prospective controlled data to support a benefit of endoscopic surveillance with respect to early cancer diagnosis or cancer-specific mortality. Given the relatively low progression rates as reported, design and completion of a statistically powered study in this regard would require a large number of patients over an extended follow-up period. A recent nested case-control study in a community-based setting, performed in a relatively closed system to permit adequacy of follow-up, did not detect evidence of reduced EAC-related mortality conferred by endoscopic surveillance among patients with BE [18].

### Initial endoscopic inspection

**Figure 1.**
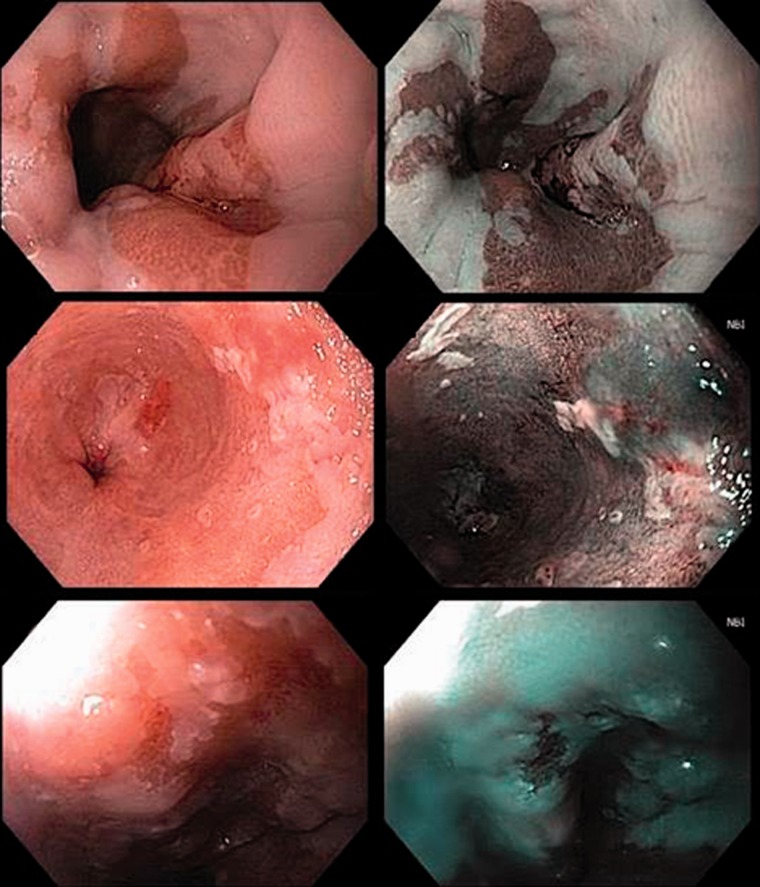
Representative white light (left panel) and NBI (narrow band imaging, right panel) images of T1a esophageal adenocarcinoma.

Advances in endoscopic technology, endoscopic diagnostic and sampling techniques, and endoscopist training have improved both initial detection and accurate staging of BE-associated neoplasia. Standard endoscope and processor technology now allow for high-definition, white-light inspection. In addition, enhanced endoscopic imaging modalities may serve as ‘red flag' techniques for identification of focal abnormalities. Among these techniques, narrow-band imaging (NBI) has been most extensively investigated in the diagnosis and assessment of BE. NBI filters white light to two wavelengths (450 nm and 514 nm) specific for hemoglobin absorption, thereby accentuating the mucosal vasculature (Figure 1). Studies have demonstrated a high sensitivity of NBI for detection of BE dysplasia [19], as well as the ability of NBI to detect dysplasia in a higher proportion of patients and with fewer biopsies, when compared with white light inspection [20].

NBI is a proprietary imaging technology. Competing endoscope manufacturers have developed additional proprietary endoscope/processor technologies with the intention of enhancing mucosal visualization and inspection. Advanced multimodal imaging may emerge as a standard and recommended practice. ‘Tri-modal' imaging, including high-resolution endoscopy, NBI, and autofluorescence imaging, may result in enhanced detection of neoplasia [21, 22], although not to a degree sufficient to allow elimination of the need for random or targeted mucosal tissue sampling [22].

Irrespective of the imaging modality employed, emphasis must be placed on detailed endoscopic inspection of the BE segment prior to biopsy sampling. The ability to detect BE-associated neoplasia may be influenced by inspection variables unrelated to endoscopic tools or technology. Akin to colonoscope withdrawal time for colonic polyp detection, the ability to detect dysplasia within a BE segment may correlate with endoscopic inspection time. In one study, endoscopists who spent an average of more than one minute inspecting each centimeter of the BE segment length were more likely to detect suspicious lesions than those devoting less time [23]. Also, several studies have demonstrated that the directional distribution of BE-associated neoplasia is not uniform, but favors certain locations within the esophageal circumference [24–26]. With the patient in the left lateral decubitus position during endoscopy, a majority of neoplastic lesions may be identified between the 1 o’clock and 5 o’clock locations in individuals with both short-segment and long-segment BE [26].

When proceeding to biopsy, appropriate endoscopic sampling of a BE segment consists of initial biopsy of visible lesions such as a nodule, ulcer, or other focal abnormality, followed by systematic four-quadrant biopsies every 1–2 cm along the length of the Barrett’s segment. Biopsies from each focal abnormality or anatomic level should be placed and labeled in separate specimen containers, in order to permit localization of disease and facilitate either future repeat sampling or targeted endoscopic resection; however, optimal endoscopic sampling of BE may face hurdles in widespread practice. Data from a United States pathology database suggest that patients with BE may not undergo a sufficient number of biopsies, and that this deficiency may become more pronounced with increasing length of the BE segment [27].

### Endoscopic staging of mucosal neoplasia

Histopathological grading of BE includes IM without dysplasia, IM containing low-grade dysplasia (LGD), and IM containing high-grade dysplasia (HGD). HGD is now synonymous with tumor *in situ* (Tis) according to staging guidelines from the American Joint Committee on Cancer (AJCC). Progression through these stages prior to development of EAC may not necessarily be unidirectional (i.e. spontaneous regression may occur) and may not be stochastic or sequential. Given an estimated annual progression rate from HGD to EAC of at least 6–7% [28], confirmed HGD has historically served as an actionable diagnosis prompting therapeutic intervention.

Expert histopathological review should be performed in all cases where biopsies detect dysplasia. In cases when biopsies are indefinite for dysplasia, a repeat endoscopy with biopsies should be performed within 6 months. If no dysplasia is detected on this subsequent examination, the frequency of future surveillance should be performed at an interval appropriate to non-dysplastic BE. A surveillance strategy for LGD should consist of repeat endoscopy with biopsies at 6-month intervals (see Table 1).

**Table 1.** Guidelines for screening and surveillance of Barrett’s esophagus

	Year	Screening	Surveillance
American College of Gastroenterology	2008	No recommendation for or against	No dysplasia: 3 years LGD: 1 year HGD without endoscopic therapy: 3 months
American Gastroenterological Association	2011	Recommended for patients with multiple risk factors for EAC Recommended against for general population with GERD	No dysplasia: 3–5 years LGD: 6–12 months HGD without endoscopic therapy: 3 months
British Society of Gastroenterology	2013	Consider in patients with chronic GERD symptoms and multiple risk factors for EAC Not justified for general population with GERD	No dysplasia and BE length <3 cm: 3–5 years No dysplasia and BE length ≥3 cm: 2–3 years LGD: 6 months

EAC = esophageal adenocarcinoma, GERD = gastroesophageal reflux disease, HGD = high-grade dysplasia, LGD = low-grade dysplasia

If biopsies detect the presence of confirmed HGD, additional investigation is necessary to exclude the presence of more advanced pathology. Historical studies of cohorts of patients undergoing esophagectomy for BE containing HGD reported occult prevalent cancer in greater than 30% of resection specimens. Such data emphasize the potential challenges of accurate disease staging attributable to several factors. The distribution of dysplasia within a BE segment is typically heterogeneous [29], resulting in the potential for biopsy sampling error and failure to detect dysplasia, even with systematic biopsy protocols. Additionally, histopathological assessment of dysplasia is subject to variable interpretation, particularly by non-expert pathologists. In a survey of community-based pathologists, only 30% correctly identified HGD—with 50% interpreting HGD as some less advanced degree of dysplasia, and 20% interpreting HGD as invasive cancer [30]. Yet, by the same token, such elevated estimates of occult cancer among patients with HGD represent data from a bygone time. More recent data—from the current, endoscopic era—suggest a low prevalence of submucosal invasive cancer among patients undergoing esophagectomy for HGD or intramucosal carcinoma [31]. Endoscopic ultrasound (EUS) can detect submucosal invasion and/or lymph node involvement in such patients [32].

Endoscopic mucosal resection (EMR) has become a valuable tool for staging of mucosal disease. Via EMR, *en bloc* mucosal resections up to 2 cm in size may be achieved via a cap-and-band or cap-and-snare-assisted technique. Such specimens provide a robust specimen for histopathological analysis, both by sampling a considerably larger mucosal surface area than forceps biopsies and reducing the potential for sampling error, and by achieving excisional depth sufficient to discriminate between mucosal and submucosal disease involvement. The latter is a point of critical emphasis in selecting patients with T1 cancer appropriate for endoscopic therapy. For T1a disease (carcinoma confined to the mucosa), the likelihood of mediastinal lymph node involvement is less than 2% [33]. Patients with T1a disease may therefore be expected to achieve remission of disease with an effective endoscopic mucosal eradication therapy. The likelihood of lymph node involvement is considerably higher—perhaps at least 30%—in individuals with T1b disease (carcinoma invasive to the submucosa) [34]. As such, embarking on endoscopic therapy for patients with T1b disease may be a more hazardous undertaking if the explicit goal of therapy is long-term cancer remission or ‘cure'.

EMR is at present the most reliable endoscopic technique for distinguishing between HGD, T1a cancer, and T1b cancer. Studies have demonstrated that EMR alters the diagnosis, compared with that rendered by forceps biopsies, in approximately 50% of patients referred for endoscopic therapy of BE-associated neoplasia, either by up-staging to a more advanced or down-staging to less-advanced pathology [35, 36]. Current expert recommendations therefore endorse EMR as essential for evaluation of HGD associated with a visible endoscopic abnormality [2, 37].

## ENDOSCOPIC ERADICATION THERAPY FOR INTRAMUCOSAL NEOPLASIA

Multiple modalities may be employed for endoscopic eradication of BE. EMR, in addition to its value as detailed above for focal excision of neoplasia and disease staging, has been utilized for wide-field or complete BE excision [38]. High rates of disease eradication may be achieved using this technique, although the post-treatment stricture rate exceeds 40%, even when performed in stepwise fashion [38, 39]. Whether the technique of endoscopic submucosal dissection (ESD)—as widely practiced in Asia—offers an advantage over EMR for therapy of BE neoplasia is uncertain [40]. It is worth emphasizing that both EMR and ESD, in contrast to all non-resection endoscopic therapies, offer a valuable specimen for histopathological analysis at the time of treatment.

Among ablative modalities, photodynamic therapy (PDT) was the first supported by rigorous controlled data demonstrating efficacy in treatment of BE neoplasia. In a landmark study of patients with BE containing HGD, randomized to either porfimer sodium PDT plus omeprazole or to omeprazole alone, eradication of HGD at 5-year follow-up was achieved in 77% of those treated with PDT plus omeprazole and 39% of those treated with omeprazole alone. Progression to esophageal cancer at 5-year follow-up was 15% in the PDT plus omeprazole arm and 29% in the omeprazole-only arm [41].

These data established porfimer sodium PDT as a viable alternative to esophagectomy, particularly among individuals who are not surgical candidates – whether due to advanced age, comorbid illness, or preference against surgical esophagectomy. Comparative retrospective data on patients undergoing PDT *versus* surgical esophagectomy for BE containing HGD at a high-volume expert center demonstrated comparable overall- and cancer-free survival over a median 5 years of follow-up [42]. The limitations of porfimer sodium include the cost of the intravenous agent, prolonged period (weeks) of photosensitivity following exposure, and an appreciable post-treatment stricture rate. The use of 5-aminolevulinic acid, an alternative oral photosensitizer, never gained widespread acceptance in the United States.

Radiofrequency ablation (RFA) was assessed for the treatment of BE dysplasia in the AIM-Dysplasia trial, in which patients were randomized to RFA plus omeprazole, *versus* sham plus omeprazole. Among patients with BE HGD, remission of dysplasia and remission of all intestinal metaplasia were achieved in 81% and 77%, respectively, at 12-month follow-up. Progression from HGD to cancer within the same time frame was observed in 2.4% (one subject) in the RFA arm compared with 19% in the sham arm [43]. Durable remission of BE in this cohort has been reported at a follow-up in interval of 3 years, with a post-treatment stricture rate of 7.6% [44].

**Figure 2.**
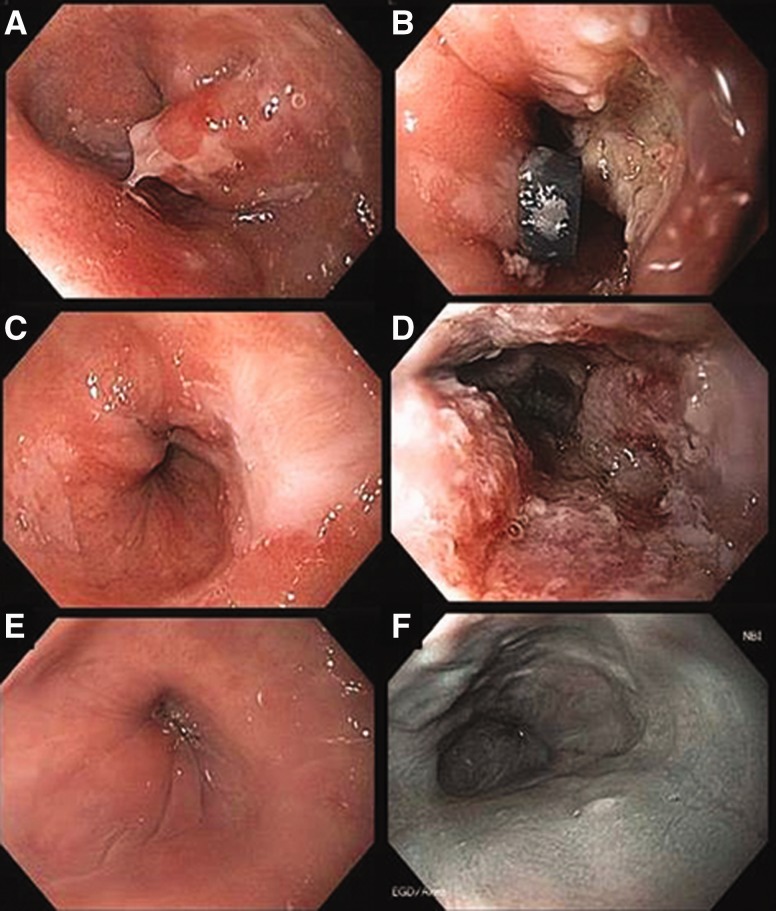
Sequential endoscopic therapy for T1a adenocarcinoma. A: T1a esophageal adenocarcinoma within Barrett s esophagus segment. B: Status following endoscopic mucosal resection (EMR). C: Three-month follow-up: squamous ingrowth at EMR site. D: Three-month follow-up: status following radiofrequency ablation (RFA). E: White light imaging: 36-month follow-up, status following two additional RFA and one additional EMR treatments. F: Narrow band imaging: 36-month follow-up, status following two additional RFA and one additional EMR treatments.

Recurrence of BE and metachronous neoplasia have been reported following endoscopic eradication therapy, underlining the importance of continued, post-treatment, endoscopic surveillance. Recurrence of neoplasia appears to be higher in individuals who do not achieve full eradication of BE [45], indicating that the optimal treatment endpoint following endoscopic therapy should be not only treatment of dysplasia/neoplasia, but also elimination of all intestinal metaplasia (Figure 2).

Given the reported efficacy of endoscopic techniques, the use of endoscopic eradication therapy as a first-line treatment for BE mucosal neoplasia has expanded considerably. The use of endoscopic therapy in the United States is estimated to have increased from 3% of cases of BE containing HGD or T1 adenocarcinoma in 1998 to 29% of such patients in 2009 [46]. Current treatment guidelines assert that most patients with BE HGD can be successfully treated with endoscopic therapy [2], which should be preferred over surgery [37]. A recent study of 1000 consecutive patients with BE and T1a cancer treated with endoscopic therapy including endoscopic resection reported a 96.3% complete response rate. Out of 140 metachronous lesions detected during follow-up, 115 were successfully treated endoscopically. Twelve patients required surgery for failed endoscopic therapy. The overall survival rate was 91% at 5 years and 75% at 10 years, with only two deaths related to esophageal cancer [47].

Additional endoscopic modalities studied for treatment of BE containing dysplasia include multipolar electrocoagulation, argon plasma coagulation, and more recently, cryoablation. Current AGA guidelines cite insufficient data to assess the ability of cryotherapy to achieve durable remission of intestinal metaplasia following endosocopic cryotherapy for BE [2].

### Endoscopic eradication therapy for less-advanced pathology

Given the reported efficacy and safety of RFA in particular, some have suggested that use of endoscopic eradication therapy may offer an opportunity to intervene in patients with pathology less advanced than HGD. Such an approach would allow for treatment of an expanded pool of at-risk patients earlier in the process of neoplastic progression [48, 49]. Studies describing RFA for the treatment of non-dysplastic BE reported complete remission of intestinal metaplasia in more than 75% of patients, with no serious adverse events [50, 51].

Current guidelines from the AGA suggest that endoscopic therapy with RFA should be an option for patients with confirmed LGD, and also for select individuals with non-dysplastic BE considered to be at risk for neoplastic progression [2]. The critical metric in determining the benefit of such intervention is careful estimation of the likelihood of disease progression. Revised estimates of the likelihood of progression from non-dysplastic BE to adenocarcinoma— from 0.5–0.3%, or perhaps as low as 0.12% per year [13–15] significantly influence the number needed to treat to prevent one case of cancer. Future ability to stratify patients into those with and without risk of neoplastic progression may help to further target therapy to a subgroup of patients at higher perceived risk.

An expert group in Amsterdam has demonstrated that confirmed LGD may have a progression rate to the combined endpoint of HGD/cancer of 13.4% per year [52]. A recent randomized trial by this group comparing RFA *versus* endoscopic surveillance for LGD reported progression at 3 years to the combined endpoint of HGD/cancer of 1.5% in the ablation arm and 26.5% in the surveillance arm [53]. Alternatively, a recent meta-analysis of 2694 patients reported a considerably lower progression rate from LGD to the combined endpoint of HGD/cancer of 1.73% per year, weighed against an annual mortality unrelated to esophageal disease of 4.7% [54].

## WHAT ABOUT SUBSQUAMOUS INTESTINAL METAPLASIA?

Subsquamous intestinal metaplasia (SSIM), colloquially referred to as ‘buried Barrett’s', describes glandular epithelium beneath overlying squamous mucosa. SSIM is in principle not visible by endoscopic luminal inspection, and is detectable only by imaging or tissue sampling to the level of the *lamina propria*. There are reports of neoplasia arising from SSIM following endoscopic therapy [55], and concerns have therefore been raised that (i) endoscopic therapy may influence development of SSIM and that (ii) neoplasia arising from SSIM may elude standard endoscopic surveillance.

SSIM may develop below islands of squamous mucosa following chronic pharmacological acid suppression and is therefore highly prevalent among BE patients naïve to endoscopic therapy. As a consequence of relative protection from exposure to luminal gastric and bile acid, SSIM may have distinct biological properties and, in theory, a lower malignant potential in comparison to surface BE. Endoscopic ablation therapy does not appear to accelerate the development of SSIM. In fact, the prevalence of SSIM may decrease following RFA—although forceps biopsies may not be of sufficient depth to routinely capture *lamina propria* necessary to assess for SSIM, and this may be particularly the case following RFA [56].

**Table 2.** Recommendations for endoscopic eradication therapy in Barrett’s esophagus

	Year	HGD	LGD	Non-dysplastic IM
American College of Gastroenterology	2008	Endoscopic ablation or surgical esophagectomy	No recommendation	No recommendation
American Gastroenterological Association	2011	Endoscopic therapy with EMR, PDT, or RFA	RFA is a therapeutic option	RFA ( ± EMR) for select individuals at risk for progression
British Society of Gastroenterology	2013	Endoscopic therapy preferred over esophagectomy	Not routinely recommended	No recommendation

EMR = endoscopic mucosal resection; HGD = high-grade dysplasia, IM = intestinal metaplasia, LGD = low-grade dysplasia, PDT = photodynamic therapy, RFA = radiofrequency ablation

While further investigation and long-term follow-up is necessary to better understand SSIM, concern regarding SSIM should not at this time influence standard endoscopic inspection and diagnosis of SSIM or selection of patients with BE for endoscopic therapy.

## FUTURE DEVELOPMENTS

Current advances in our understanding of the natural history and management of BE have created a window of opportunity. Whether or not endoscopy can achieve the ultimate goal in BE management—reduction in EAC incidence and mortality—may further depend upon the extent to which future developments in biomarkers and disruptive technology can be incorporated into clinical practice.

From the standpoint of screening and diagnosis, minimally invasive endoscopy (i.e. unsedated transnasal endoscopy) or non-endoscopic screening technologies may allow for greater more widespread application of cost-effective screening to individuals at risk [57].

Among individuals diagnosed with BE, novel biomarkers capable of stratifying those likely to progress to dysplasia/cancer from likely non-progressors may allow for selective application of endoscopic surveillance and therapy. Technologies capable of ‘optical biopsy', including confocal laser endomicroscopy or peptide-based imaging [58, 59], may afford opportunities for real-time histopathological diagnosis at point of care to triage immediate application of therapy. Confocal laser endomicroscopy, via either a probe-based or endoscope-based device, in conjunction with intravenous administration of fluoroscein, allows for real-time visualization of cellular architecture. A recent multicenter, randomized trial demonstrated that the combination of high-definition white light endoscopy, confocal endomicroscopy, and targeted biopsies led to increased diagnostic yield for dysplasia with fewer biopsies compared with high definition white light endoscopy and random biopsies [58]. Optical coherence tomography-based imaging platforms, such as volumetric laser endomicroscopy, capable of mapping an entire Barrett’s segment including sub-epithelial structures, may eliminate the potential for inspection bias or sampling error, and potentially even allow automated application of targeted therapy [60].

## SUMMARY

The entities of BE and BE-associated neoplasia, considered from the perspective of both public health and patient-based challenges, are poised at the junction of two divergent trends. On the one hand, the incidence of EAC continues to escalate despite current endoscopic practice. On the other, endoscopic imaging technologies are becoming increasingly powerful in their ability to help endoscopists recognize disease, and endoscopic mucosal resection and ablation options have altered the treatment landscape and offered unprecedented opportunities for intervention for patients with BE-associated neoplasia.

Endoscopic screening and surveillance strategies have become the norm in western endoscopic practice, and are likely to remain so despite current lack of data to support their effectiveness. Also, despite the ability of endoscopy to accurately diagnose and stage BE, current endoscopic and scientific technologies fail to account for asymptomatic individuals at risk and, among patients diagnosed with BE, fail to predict which individuals are at risk for progression. While tremendous investigative and technological progress has been made over the past decade to inform current treatment approaches, acquisition of data from further controlled studies and development of risk stratification algorithms will influence whether endoscopic therapy can be sensibly applied to an expanded population of patients with BE earlier in the disease process. Additionally, investigators and clinicians will need to be open to change and think creatively ‘outside the box' in their approach to BE screening, surveillance and treatment, in order to capitalize on opportunities for further progress towards the ultimate goal of reducing mortality from EAC.

**Funding:** This work was supported by the National Institutes of Health: grant R01CA140574 to Dr. Hur.

**Conflict of interest:** none declared.
